# “Monocytes in B-Cell Malignancies: Their Role in Disease Progression and Therapy Resistance”

**DOI:** 10.1007/s11912-025-01732-9

**Published:** 2025-11-12

**Authors:** Katsiaryna Marhelava, Justyna Jakubowska, Agata Pastorczak, Malgorzata Firczuk

**Affiliations:** 1 Department of Immunology, Mossakowski Medical Research Institute, Polish Academy of Sciences, 5 Pawinskiego Str, Warsaw, 02-106 Poland; 2 Department of Pediatrics, Oncology and Hematology, Medical University of Lodz, Lodz, Poland; 3 Department of Genetic Predisposition to Cancer, Medical University of Lodz, Lodz, Poland; 4 Department of Immunology, Medical University of Warsaw, Warsaw, Poland

**Keywords:** Monocytes, B-cell malignancies, Tumor microenvironment, Therapy resistance, Therapeutic strategies

## Abstract

**Purpose of Review:**

Although current treatments have improved outcomes in B-cell malignancies, therapy resistance remains a major challenge and is often driven by the tumor microenvironment. The purpose of this review was to assess the roles of monocytes and monocyte-derived cells in leukemia and lymphoma and to evaluate the potential of therapies targeting these populations.

**Recent Findings:**

Recent studies indicate that monocytes and monocyte-derived cells are associated with poor prognosis, therapy resistance, and treatment-related side effects in B-cell malignancies. These cells can suppress anti-tumor immunity, support malignant cell survival, and impair therapeutic efficacy. Strategies to deplete or reprogram these populations have shown promise in restoring immune function and enhancing the effectiveness of current treatments.

**Summary:**

Targeting suppressive monocyte-derived populations offers a promising strategy to overcome therapy resistance and improve outcomes in B-cell malignancies. Modulating these cells may reduce relapses, enhance treatment responses, and provide a foundation for the development of next-generation immunotherapies. Nevertheless, further studies are needed to better define the immunosuppressive and therapy-relevant subpopulations in specific diseases, which will be critical to translating these strategies into effective clinical interventions.

## Introduction

B-cell malignancies comprise a diverse group of hematologic cancers that arise from B cells at various stages of differentiation. B-cell acute lymphoblastic leukemia (B-ALL) originates from immature B-cell precursors that undergo uncontrolled proliferation and accumulate predominantly in bone marrow (BM) and peripheral blood (PB). In contrast, chronic lymphocytic leukemia (CLL) and most mature B-cell lymphomas—including follicular lymphoma (FL), diffuse large B-cell lymphoma (DLBCL), and mantle cell lymphoma (MCL)—derive from more differentiated B cells. These mature malignant B cells primarily reside in secondary lymphoid organs such as lymph nodes and the spleen, although CLL cells also circulate extensively in peripheral blood.

Current frontline treatments for B-cell malignancies typically involve multi-agent chemotherapy regimens combined with monoclonal antibodies that target B-cell surface markers, mainly CD20 (e.g., rituximab, obinutuzumab). Despite high initial response rates, some patients experience relapses or refractory disease. To address these challenges, recent advances have introduced novel immunotherapies such as bispecific T-cell engager antibodies (BiTEs) that redirect cytotoxic T cells to malignant B cells, and chimeric antigen receptor T-cell (CAR-T) therapies, which genetically engineer patients’ T cells to recognize and eradicate tumor cells. These approaches have shown significant efficacy in relapsed or refractory cases, transforming the therapeutic landscape of B-cell malignancies.

The tumor microenvironment (TME) in B-cell malignancies is a complex and dynamic niche where malignant cells interact with various immune and stromal cells. Among these, monocytes and their derivatives play a crucial role. Monocytes are innate immune cells characterized by functional plasticity and the capacity to respond to environmental signals. In the context of malignancy, they can be reprogrammed by tumor-derived factors to adopt predominantly tumor-promoting phenotypes. This reprogramming often supports immune suppression, facilitates tumor growth, and contributes to resistance against both conventional chemotherapy and modern immunotherapies. Importantly, elevated levels or altered subsets of monocytes have been associated with poor prognosis in several B-cell malignancies, underscoring their clinical relevance. As a result, monocytes have emerged as potential therapeutic targets, with growing interest in strategies aimed at reprogramming their function or selectively eliminating immunosuppressive populations.

This review addresses monocyte biology and function both under physiological conditions and within the context of B-cell malignancies. We explore how monocytes are recruited, reprogrammed, and shape the leukemia and lymphoma microenvironments in B-ALL, CLL, and B-cell lymphomas. In addition, we address current methods for monocyte detection and characterization, with a focus on flow cytometry. Furthermore, we discuss the implications of monocyte–tumor interactions for prognosis and treatment, highlighting emerging therapeutic approaches that target monocytes to improve clinical outcomes.

## Monocytes in Physiological Conditions

### Development and Phenotype of Monocytes

Monocyte development in BM follows a tightly regulated, hierarchical differentiation pathway. It begins with hematopoietic stem cells (HSC), which give rise to multipotent progenitors (MPP). MPP subsequently commit to the myeloid lineage via common myeloid progenitors (CMP), which differentiate into granulocyte-monocyte progenitors (GMP) and monocyte-dendritic cell progenitors (MDP), each contributing to distinct immune cell subsets [1]. Once formed, monocytes are released into the bloodstream [2], comprising 10% of the peripheral leukocytes in humans and around 4% in mice [3]. Research on monocyte heterogeneity has revealed considerable diversity [4–7], yet the prevailing nomenclature defines three main subsets based on expression of the bacterial endotoxin receptor CD14 and the low-affinity receptor for immunoglobulin G - CD16. Under physiological conditions, classical monocytes (CD14^high^CD16^−^; CM) represent ~ 85% of the population, non-classical monocytes (CD14^low^CD16^+^; NCM) ~ 10%, and intermediate monocytes (CD14^high^ CD16^+^; ITM) ~ 5% [8]. CM correspond to mouse Ly6C^high^/CCR2^+^/CD62L^+^/CD43^low^, whereas the NCM resemble mouse Ly6C^low^/CCR2^low^/CD62L^−^/CD43^+^ monocytes [9].

Current evidence suggests that CM can undergo a sequential differentiation in PB, transitioning first into ITM and eventually into NCM [2, 10]. However, several specific proteins that are uniquely regulated in intermediate monocytes have been identified, challenging the notion of these cells being purely transitional [11]. Despite this outstanding protein profile, ITM are shown to be more similar to NCM than the classical subset [12].

Monocyte subset distribution is stable under homeostasis but shifts in response to various physiological and pathological stimuli. In the context of systemic infections such as sepsis, the proportion of CD16⁺ monocytes can rise temporarily [13] and NCM were found to expand with age [14, 15]. Moreover, extrinsic factors including circadian fluctuations, acute psychological stress, and nutritional status have been shown to modulate monocyte subset distribution [16–18]. Such changes reflect not only cell numbers but also alterations in gene expression and metabolism, directing immune responses toward pro-inflammatory or immunosuppressive states depending on context.

### Techniques for Monocytes Detection and Characterization

Accurate detection and characterization of monocyte subsets is essential for understanding their physiological roles and dysfunctions in cancer. Direct assessment in whole blood is often recommended to preserve the native monocyte phenotype. However, monocyte isolation may be necessary for functional assays or sequencing studies. Isolation methods each have trade-offs, affecting purity, marker expression, and function. Density gradient isolation of PBMC often skews subset proportions, typically resulting in decrease in number of CM and enrichment of NCM, alongside upregulation of CD16 expression [19]. CD14-positive selection yields high purity [20] but can mask epitopes, complicating immunophenotyping, and may activate monocytes, altering gene expression and cell behavior. Negative magnetic selection preserves functional integrity of cells but typically achieves lower purity [21].

Techniques such as immunohistochemistry (IHC), single-cell RNA sequencing (scRNA-seq), and flow cytometry have been applied to characterize monocytes and monocyte-derived cells. IHC on fixed tissue sections enables spatial localization of monocytes and macrophages and investigation of their interactions with other cells in bone marrow and lymph nodes [22]. The major limitation of IHC is the small number of markers that can be assessed simultaneously. In contrast, scRNA-seq offers high-resolution insights into monocyte heterogeneity and dynamic gene expression across conditions like inflammation, cancer, sepsis, and infection [5, 23, 24], but is costly, technically demanding, and RNA levels do not always reflect protein abundance or functional state. High-dimensional flow cytometry can analyze multiple markers simultaneously, providing rapid, comprehensive phenotyping and is often the method of choice [25]. Therefore, the subsequent sections will focus mainly on flow cytometry.

**Fig. 1:**
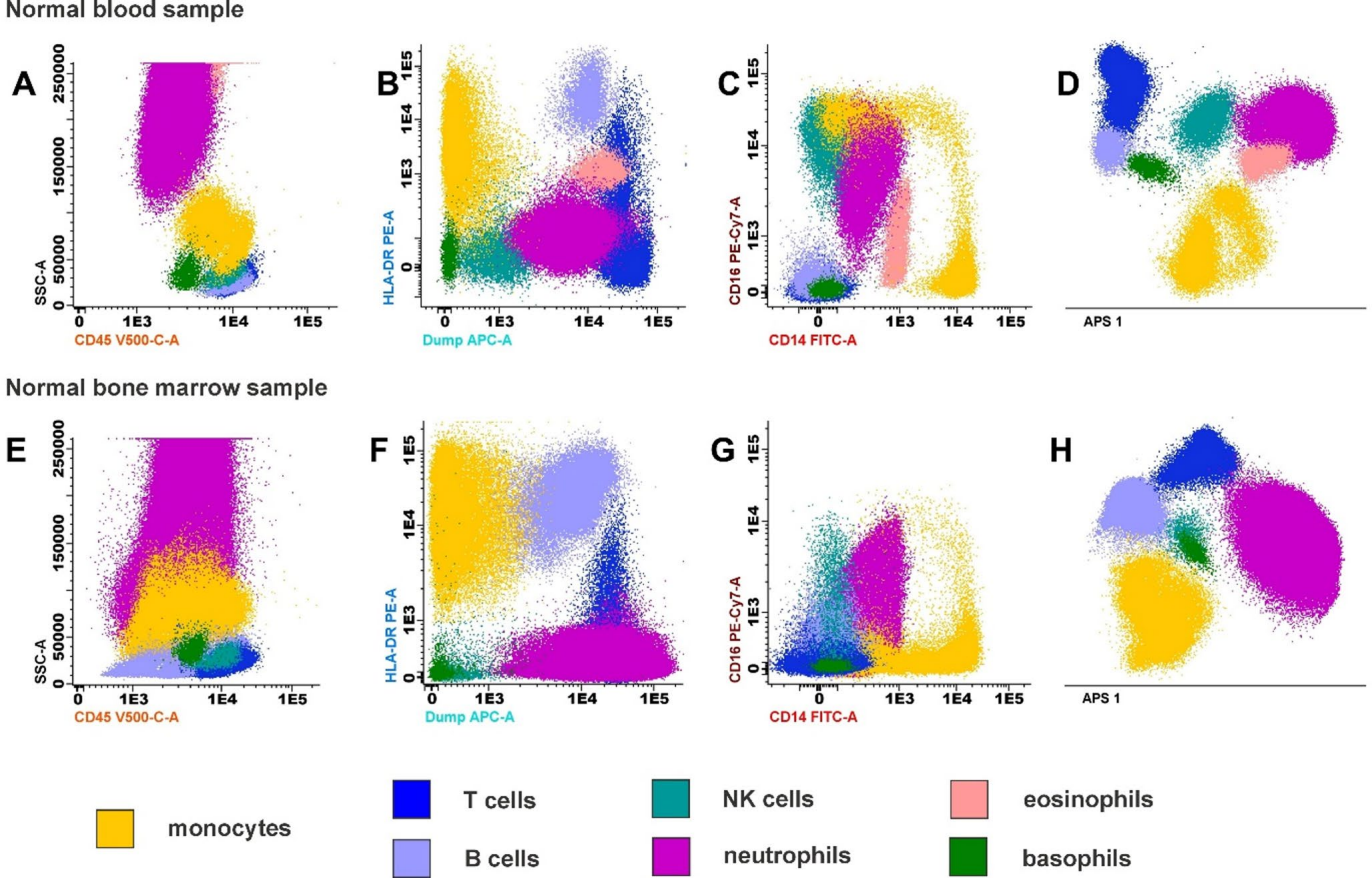
Multidimensional flow cytometry analysis enables improved monocyte identification across sample types. Representative flow cytometry plots from normal peripheral blood (**A-D**) and normal bone marrow (E-H) illustrate the challenges of accurately identifying monocyte subsets using conventional gating strategies (**A-C**,**E-G**) due to marker overlap with other cell populations. Unlike traditional 2D dot plots, which evaluate two parameters at a time, Automatic Population Separator (**D**,**H**) analyzes all parameters simultaneously, improving discrimination of overlapping populations. Despite this, identification of CM, ITM, and NCM remains challenging and depends on marker resolution (**C, G**). The antibody panel included leukocyte marker CD45, core monocyte markers CD14 and CD16, activation and differentiation marker HLA-DR, as well as a dump channel consisting of CD3, CD19, CD56, and CD66c for exclusion of T cells, B cells, NK cells, and granulocytes, respectively. The cells were acquired with a FACSLyric flow cytometer (BD) and analyzed with Infinicyt software (BD). Automatic Population Separator analysis was performed to visualize multidimensional separation of cell populations

Due to stable surface marker expression, analysis of monocyte subsets in PB is feasible and relatively simple to interpret. In contrast, BM samples require more nuanced, context-specific gating strategies to effectively distinguish monocytes from hematopoietic progenitor cells with overlapping phenotypes [26]. Traditional gating strategies relying on CD45, CD14, CD16, and scatter characteristics have inherent limitations and need further refinement for effective monocyte detection in patients. “Dump channels” containing lineage (Lin) markers such as CD3 (T cells), CD19/CD20 (B cells), CD56/CD2/CD7 (NK cells), CD66b (granulocytes), and myeloid dendritic cell markers (e.g., CD1c, CD141, CD88, CD89) help to effectively exclude non-monocytic populations. Consideration of additional markers, typically not expressed on lymphocytes or granulocytes under steady-state conditions, can significantly improve subset discrimination and classification (Fig. 1).

Key monocyte-specific markers include HLA-DR, Toll-like receptor 2 (TLR2/CD282), CD33, CD11b, CD36, CD11c, CD86, CD64, and CCR2 [12, 27–33]. Table 1 lists antigens, along with their advantages and limitations, that can be incorporated into antibody panels to enhance monocyte identification in the context of B-cell malignancies. As antigen expression may fluctuate under pathological or inflammatory conditions, the use of multiple pan-monocyte markers is recommended to ensure accurate gating [34, 35].

**Table 1:** Antigens used for monocyte identification in B-cell malignancies: advantages and limitations (based on [27, 29, 31, 32], [34–36])

Antigen	Advantages	Limitations
**CCR2**	High on CM and ITM; subset-specific	Overlap between CM and ITM
**CD11b**	Indicates activation; expressed on monocytes and phagocytes	Expressed on neutrophils and other myeloid cells
**CD11c**	Higher on ITM/NCM; low on lymphocytes	Shared with dendritic cells
**CD14**	Core monocyte marker; high expression on CM	Internalized upon activation; clone- and method-dependent; variable in BM
**CD16**	Enables subset discrimination; useful in inflammation profiling	Upregulated after isolation; clone variability; expressed on neutrophils and basophils
**CD300**	Expressed only on mature monocytes; better than CD86 for basophil exclusion	Not expressed on promonocytes
**CD33**	Myeloid-specific; low lymphoid expression	Downregulated in sepsis; expressed on immature NK, blasts, and basophils; expression downregulated by gemtuzumab ozogamicin (GO)
**CD36**	Expressed on all monocyte subsets	Low on NCM; expression on other myeloid cells
**CD64**	Stable myeloid marker; reliable in bone marrow	Lower on NCM; transient on immature myeloid precursors
**CD86**	Activation marker; expression on NCM	Upregulated on B-cell blasts and basophils
**CX3CR1**	High expression on NCM; efficient subset distinction	Decreased expression in inflammation; expressed on NK and some T cells
**HLA-DR**	High specificity for monocytes; excludes granulocytes and lymphocytes	Downregulated in sepsis and certain cancers; shared with some DC
**TLR2**	Monocyte-specific; low expression on lymphocytes	Expression altered by infection or stimulation

### Functions and Differentiation of Monocytes

PB monocytes coordinate both innate and adaptive immunity by phagocytosing pathogens, producing reactive oxygen species (ROS), secreting cytokines and chemokines, recruiting neutrophils, presenting antigens, and modulating lymphocyte activity.

Several genome-wide analyses provided a comprehensive definition of monocyte subsets during healthy conditions, pointing out the biggest differences in gene activity between CM and NCM. CM exhibit high expression of genes involved in driving bacterial responses, inflammation, and low-density lipoprotein uptake, whereas NCM upregulate genes linked to cytoskeletal dynamics, invasion to inflammatory tissues, and terminal differentiation [37]. ITM express genes associated with antigen presentation and proangiogenic functions and, similarly to CM, can produce proinflammatory cytokines and ROS. Interestingly, ITM are the only subset expressing CCR5, supporting their role in HIV dissemination [38]. Gene expression profiling showed that each monocyte subset contains smaller sub-populations defined by activation and differentiation, indicating that the monocyte classification is still incomplete and warrants further investigation [6, 39].

Monocyte trafficking into tissues is guided mainly by chemokines and adhesion molecules. The three subsets show distinct receptor profiles, suggesting tissue-specific recruitment: CM have high CCR2, CD62L, CD11b, and TLR-4 expression but low CX3CR1; NCM have high CX3CR1 and CXCR4 expression but low CCR2; ITM show lower levels of both CX3CR1 and CCR2 [40]. Depending on local environmental signals in the tissue coming from cytokines, growth factors, and cell-cell interactions, CM can differentiate into macrophages, dendritic cells (DC), osteoclasts, or foam cells, while NCM generate distinct macrophage or DC subsets [41]. Monocytes differentiate into macrophages in response to macrophage colony-stimulating factor (M-CSF) and IL-34 [42], whereas granulocyte-macrophage colony-stimulating factor (GM-CSF) and IL-4 drive their differentiation into DC [43, 44]. An adoptive monocyte transfer into humanized mice revealed that CM primarily gave rise to inflammatory macrophages, whereas NCM differentiated into anti-inflammatory, vasculature-patrolling cells. Transcriptomic and phenotypic profiling demonstrated that these fates are not entirely plastic but instead shaped by pre-existing subset-specific programming and contextual signals [45].

## Monocytes in Cancer

Monocyte counts vary across cancer types, and the proportion of monocyte subsets in PB is associated with inflammation, cancer type, and patient prognosis [46]. Monocytes are recruited into TME via several receptor–ligand pairs, with CD62L/CD62L ligands, CX3CR1/CX3CL1, CCR2/CCL2, and VEGFR1/VEGF-A being particularly important [47]. Within inflammatory TME, monocytes can differentiate into macrophages or DC, supporting anti-tumor immunity by promoting extracellular matrix degradation, stimulating T-cell activation, and enhancing proliferative responses [48].

However, as disease progresses, the TME becomes increasingly immunosuppressive, reshaping monocyte differentiation through cytokines, growth factors, and signaling pathways. The generation of functional DC from monocytes was shown to be impaired in several cancer types [49–51]: tumor-derived factors either block their differentiation, reduce antigen-presenting capacity, or even skew them toward immunoregulatory phenotype (regDC) with the ability to induce regulatory T cells (Treg) [52]. Consequently, most monocyte-derived cells in TME acquire immunosuppressive fates, predominantly M2-polarized tumor-associated macrophages (TAM) and monocytic myeloid-derived suppressor cells (M-MDSC). TAM are typically defined by high CD206 and CD163 expression [53], while M-MDSC are characterized by CD14 positivity and reduced HLA-DR levels [54]. M-MDSC can arise from immature or reprogrammed PB monocytes [55] and may even further differentiate into TAM subsets, linking the MDSC and macrophage compartments [56].

**Fig. 2:**
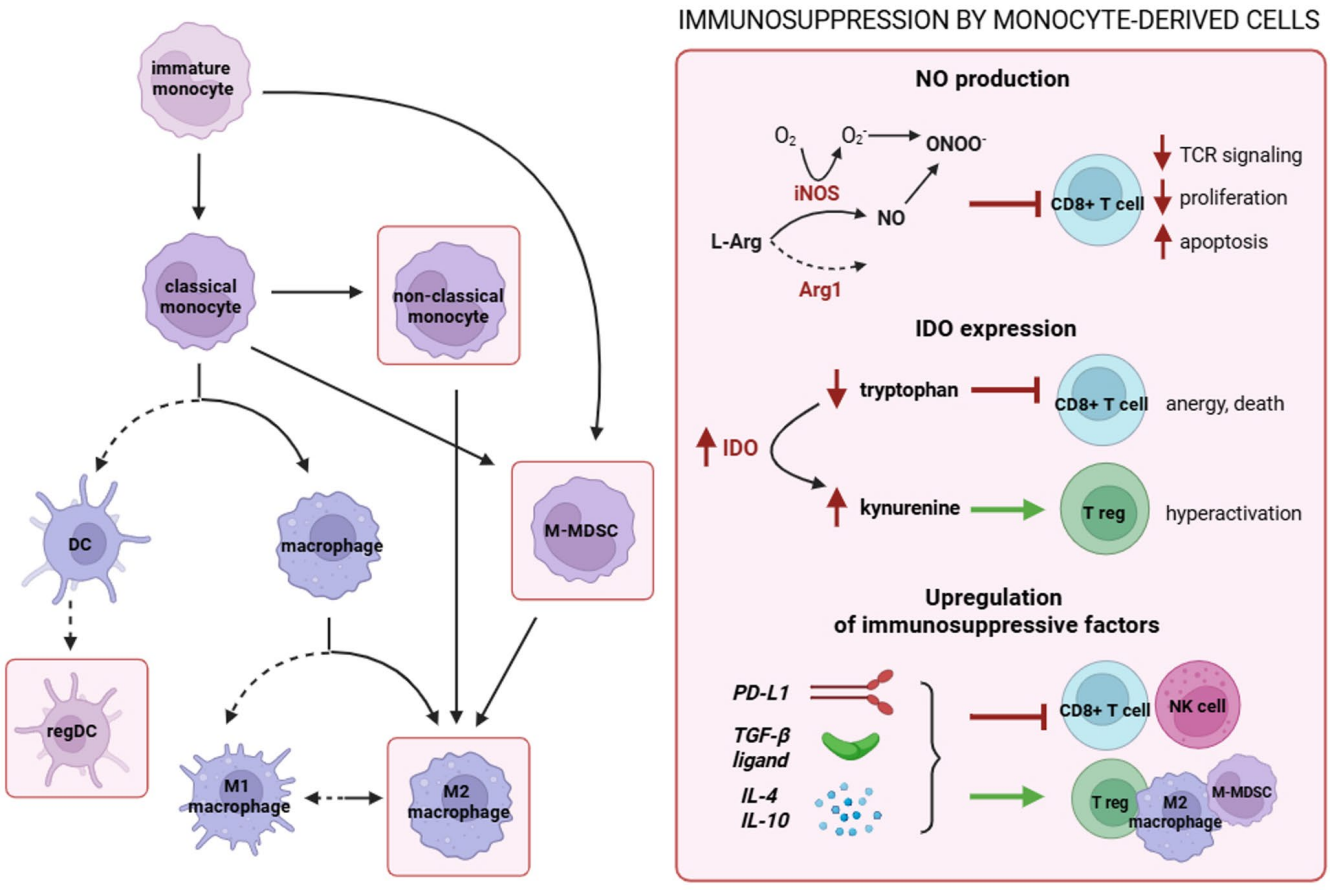
In the TME, monocytes can give rise to inflammatory, anti-tumor cells (M1 macrophages, DC), but tumor-derived signals strongly bias their differentiation toward immunosuppressive phenotypes: NCM, M-MDSC, regDC, M2 macrophages (framed in the figure). These cells suppress immunity through NO and IDO metabolism, checkpoint expression (e.g. PD-L1), and anti-inflammatory cytokines (TGF-β, IL-4, IL-10), leading to inhibition of CD8⁺ T and NK cells and reinforcement of Treg and other suppressive populations. Created in BioRender. Firczuk, M. (2025) https://BioRender.com/0rh2bwj

All immunosuppressive monocyte-derived populations, including TAM, regulatory DC, and M-MDSC, contribute to tumor immune evasion via overlapping mechanisms. These include checkpoint upregulation (e.g., PD-L1) [53, 57], secretion of immunosuppressive cytokines (IL-10, TGF-β, IL-4) [58], and metabolic regulation through nitric oxide (NO) [57] and indoleamine 2,3-dioxygenase (IDO) [59] pathways (Fig. 2).

Collectively, these processes suppress effector T-cell proliferation and cytotoxicity, promote Treg expansion, and reinforce an immunosuppressive tumor environment. Beyond immune modulation, M2 macrophages also facilitate angiogenesis and drive therapeutic resistance through activation of PI3K/AKT, JAK/STAT, MAPK, and related signaling cascades [60].

###  Monocytes in B-Cell Leukemia

#### Monocytes in B-Cell Acute Lymphoblastic Leukemia

B-ALL is a hematological malignancy characterized by the uncontrolled proliferation and accumulation of B-lymphoid precursors in BM. The TME, including myeloid cells, plays a pivotal role in this disease by supporting leukemic cell survival and promoting resistance to therapy [61].

Recent evidence points to a significant role for monocyte subset shifts in B-ALL progression and patient outcomes. Matched diagnostic PB and BM samples of 57 relapsed and 57 remission B-ALL patients revealed a significant predominance of NCM in relapse cases, whereas remission controls are enriched in CM. Moreover, a higher percentage of NCM in PB at diagnosis correlated with an increased risk of relapse in B-ALL [62]. Experimental evidence, including monocytic RNA-seq profiling, suggests that B-ALL cells promote the transition of CM into NCM, likely in response to vascular damage. While these leukemia-associated NCM did not suppress T-cell proliferation in vitro, they might promote B-ALL progression and treatment resistance, as it was shown that in mice, CSF1R-mediated monocyte depletion enhanced tyrosine kinase inhibitor efficacy [63]. In vitro, monocytes co-cultured with B-ALL cells upregulate the expression of CXCL10 chemokine, which interacts with CXCR3 on leukemic cells, enhancing their motility and invasiveness. Elevated CXCL10 levels were also observed in B-ALL-associated monocytes and patient plasma [64]. These findings suggest that monocytes contribute to leukemia progression by facilitating the dissemination of leukemia cells.

During the progression of B-ALL, monocytes are actively recruited from the PB into the BM. CCL2 and CX3CL1 chemokines are markedly increased in the B-ALL BM, promoting infiltration of both CM and NCM. The complement component C5a, which was found to be increased in the BM plasma of B-ALL patients, also contributes to monocyte recruitment as a potent chemoattractant [65]. Once recruited to the BM, monocytes can be reprogrammed by the leukemic cells to support leukemia progression. Moreover, it was shown that cytotoxic chemotherapy or treatment-associated inflammation may contribute to the expansion or persistence of immunosuppressive monocytes during early treatment. From 15 pediatric patients, 60% had increased numbers of M-MDSC at the end of induction therapy as compared to diagnosis, both in the PB and BM [66]. Immunohistochemical analyses of BM biopsies from patients with B-ALL at diagnosis and relapse have also shown increased numbers of M2 macrophages compared to healthy controls [65, 67]. Additionally, the latter study revealed that although infiltrating macrophage numbers decreased in patients in remission, they remained significantly higher than in controls, indicating persistent immunosuppressive macrophages despite clinical improvement [67]. M2 macrophages were identified as a key source of pro-leukemic FLT3 ligand (FLT3L) in the B-ALL microenvironment, where B-ALL blasts express high levels of the poor-prognosis-associated receptor FLT3. Co-culture experiments revealed that TAM-derived FLT3L enhanced B-ALL viability through activation of FLT3 and ERK signaling pathways. As FLT3 gain-of-function mutations are rare (< 5%) in B-ALL, these findings highlight ligand-driven FLT3 activation as a putative therapeutic target [68]. The process of monocyte differentiation into anti-tumor DC in the BM was observed to be impaired by leukemia-derived BMP4, a TGF-β family member that activates BMP signaling in monocytes. In vitro, this led to fewer fully differentiated DCs with an immunosuppressive phenotype, increased expression of pro-tumoral factors and reduced T-cell–stimulatory capacity. BMP pathway inhibition reversed these changes, confirming BMP4’s role in this reprogramming [69].

#### Monocytes in Chronic Lymphocytic Leukemia

CLL cells primarily accumulate in PB but proliferate within lymphoid organs such as the lymph nodes, spleen, and BM. Within these tissues, CLL cells engage in complex, bidirectional interactions with surrounding cells, including myeloid cells, mesenchymal stromal cells (MSC), T cells and NK cells. These interactions create specialized niches that support CLL cell survival, proliferation, and disease progression [70].

CLL patients exhibit a significantly higher percentage of circulating CD16⁺ monocytes compared to healthy controls, with levels directly correlating with the extent of bone erosion. Exposure of healthy monocytes to CLL-conditioned medium led to upregulation of RANK, RANKL, and notably CD16. These monocytes also displayed a marked tendency to differentiate into osteoclasts [71]. Interestingly, another study highlighted functional differences between ITM and NCM in CLL. Although both subsets were elevated in the PB of CLL patients compared to healthy donors, the proportion of NCM significantly declined with increasing disease severity. Intracellular cytokine analysis revealed that ITM were skewed toward IL-10 production, while NCM predominantly produced TNF and IL-12, suggesting a potential anti-tumor role for the latter subset [72]. Additionally, abnormally elevated levels of soluble CD14 were observed in the serum of CLL patients. In vitro, CLL cells induced the secretion of this protein in monocytes, which enhanced NF-ĸB activity and protected cancer cells from apoptosis [73]. CLL cells can also drive the conversion of normal monocytes into M-MDSC, which are expanded in the PB of untreated patients [74]. Elevated M-MDSC levels correlate with significantly shorter OS, adverse prognostic features, and reduce CD3ζ expression in T cells, underscoring their role in establishing an immunosuppressive tumor microenvironment [75].

Monocytes recruited to the CLL TME are believed to differentiate into monocyte-derived nurse-like cells (NLC), that accumulate in the lymph nodes, BM, and spleen. NLC have been shown to protect CLL cells from both spontaneous and drug-induced cell death in co-culture systems [76]. Although NLC share surface markers with monocytes, monocyte-derived DC, and macrophages, they express significantly higher levels of CD68 [77]. CD163 expression on NLC was shown to positively correlate with CLL cell proliferation in the lymph nodes. Elevated levels of soluble CD163 have been associated with adverse prognostic factors and worse patient survival [78]. NLC have also been identified as a source of Wnt5a, a molecule found at high levels in the plasma of CLL patients and linked to disease progression and venetoclax resistance [79]. Beyond chemotherapy resistance, NLC contribute to resistance against therapeutic antibodies. NLC derived from CLL patients show impaired antibody-dependent phagocytosis, a key mechanism of action for anti-CD20 antibodies [80]. Recent evidence implicates the CD47:SIRPα innate immune checkpoint in mediating this suppression, as siRNA-mediated depletion of SIRPα restored phagocytic activity [81]. Treatment with lenalidomide, which induced a pro-inflammatory profile in NLC, improved their phagocytic activity and ability to activate T-cell proliferation [82]. In a murine CLL model, where monocytes accumulating in the spleen resembled their human counterparts, the depletion of myeloid cells using liposomal clodronate suppressed disease progression and improved immune balance, highlighting therapeutic benefit [83].

### Monocytes in B-Cell Lymphoma

#### Monocytes in Follicular Lymphoma

FL is an incurable germinal center B-cell lymphoma located primarily in the lymph nodes. The lymphocyte to monocyte ratio (LMR) has been proposed as an easily determinable prognostic factor in patients with this cancer type. In a study including data from 100 FL patients, lower LMR after first-line therapy was associated with shorter OS and progression-free survival (PFS) [84]. A larger study of 1018 previously untreated patients with high tumor burden at diagnosis confirmed these findings [85]. The prognostic value of LMR in follicular lymphoma may reflect the pivotal role of monocytes within the TME, highlighting their contribution to disease progression.

FL malignant B cells critically depend on complex interactions within their microenvironment. The FL TME is notably enriched in exhausted cytotoxic T cells, Treg, and follicular helper T cells that support malignant B-cells. Stromal cells further contribute by deregulating chemokines and altering the extracellular matrix [86, 87]. FL-derived MSC recruit monocytes through elevated CCL2 production and drive their differentiation toward immunosuppressive M2 macrophages [88]. DC further reinforce this process by secreting CCL2 and CSF-1, the latter increasing in patient serum with disease progression. Blockade of CSF-1R on M2 macrophages was shown to reprogram them toward an anti-tumor M1 phenotype [89]. Single-cell mass cytometry of lymph node samples confirmed that at disease progression, CD163⁺ macrophages expressing PD-L1/PD-L2 increase, CD8⁺ T cells decline, and FL cells interact more closely with macrophages than with Treg or cytotoxic T cells [90]. These expanding TAM drive malignant B-cell growth together with FL-derived MSC, as supported by in vitro experiments [88], likely through elevated IL-15 production, which is essential for B-cell proliferation [91]. The positive correlation between the increase of TAM and microvascular density was also observed, confirming the pro-angiogenic role played by TAM in the TME of lymphomas [92]. Moreover, TAM might promote FL cells dissemination by inducing in them expression of several genes related to migration or invasion [89]. TAM abundance is generally linked to poor prognosis in FL. However, with the introduction of novel therapies the role of macrophages has been observed to be treatment-dependent, with their prognostic impact varying across therapeutic regimens [93]. In particular, high CD68^+^ TAM density influenced PFS favorably in the subgroup of patients with rituximab-containing treatment [94].

#### Monocytes in Diffuse Large B-Cell Lymphoma

DLBCL is an aggressive and the most prevalent form of lymphoid cancer. In certain cases, DLBCL cells can survive and proliferate independently, whereas in others, they depend on signals and support from surrounding non-malignant cells [95].

Several studies have highlighted the prognostic value of the LMR in DLBCL patients treated with immunochemotherapy, showing that low LMR at diagnosis is associated with adverse clinical features, higher risk of disease progression and shorter OS [96, 97]. Moreover, the LMR may help guide therapy in patients considered for curative-intent strategies such as CAR-T cell therapy or bispecific antibodies [98], which fail in ~ 50% of cases. In a study including patients with r/r DLBCL undergoing CAR T-cell therapy, high monocyte counts at the time of leukapheresis were associated with poorer PFS and treatment response [99]. It was shown that myeloid cells can limit CAR T-cell activation, expansion, and transduction efficacy [100]. Activated monocytes also secrete inflammatory mediators that recruit innate immune effectors contributing to cytokine release syndrome (CRS) and neurotoxicity [101], both detailed in a subsequent section.

While in many studies in DLBCL monocytes have been considered a single population, their functional heterogeneity suggests distinct roles for individual subsets. A study analyzing PB samples from 46 B-NHL/DLBCL patients found that elevated CD16^+^ monocyte levels (ITM and NCM) were associated with poor PFS. In vitro, CD16^+^ monocytes co-cultured with tumor cells and rituximab upregulated PD-L1 after phagocytosis [102]. In a more detailed analysis, monocyte subtypes were examined in 91 newly diagnosed DLBCL patients and a higher proportion and absolute count of CD16^+^ NCM - but not ITM - were associated with poor prognosis in two independent cohorts. NCM exhibited gene signatures linked to tumor growth, immune inhibition, and chemotaxis. Moreover, tumor-conditioned monocytes from healthy donors acquired an NCM-like phenotype and displayed enhanced migration toward CCL5/CXCL12, suggesting recruitment into the tumor-associated macrophage pool [103]. On the other hand, it is known that coculture of CD14^+^ monocytes with B cells from peripheral blood or from DLBCL enabled prolonged B cell survival and stimulated their proliferation [104]. IL-10, which is increased in the serum of patients with B-cell NHL, including DLBCL, and is produced by lymphoma cells, was shown to decrease HLA-DR expression on monocytes, and IL-10-induced CD14^+^HLA-DR^low^ cells inhibited the activation and proliferation of T cells [105]. M-MDSC have long been recognized for their role in promoting B-NHL development through systemic immune suppression [106]. A clinical trial with lenalidomide and R-GDP (rituximab plus gemcitabine, cisplatin and dexamethasone) conducted in patients with r/r DLBCL showed significant negative association between MDSC abundance and survival [107].

Monocytes are recruited into the lymph node TME in DLBCL [108] and this process was observed to increase with the disease progression [109]. It is driven by lymphoma-derived IL-16 [109] and additionally CCL5 chemokine. Interestingly, this CCL5-driven recruitment is a post-transformation event, absent in healthy B cells and unrelated to molecular subtypes. Single-cell analysis showed that TAM accumulated through this mechanism exhibit a noncanonical polarization profile, co-expressing both M1 and M2 markers [110], which might explain why their prognostic significance was for a long time controversial, with studies reporting conflicting outcomes regarding their association with patient survival [111]. In a recent comparative study of immune infiltrates across lymphoma subtypes, DLBCL uniquely exhibited a high infiltration of PD-L1^+^ macrophages in the lymph nodes, comprising up to 70% of total cellularity, whereas normal lymph nodes contained very few macrophages. PDL1 expression on macrophages was associated with shorter PFS and identified as a negative prognostic factor in DLBCL [112]. Additionally, TAM from the tumor tissues of R/R DLBCL patients were found to express significantly higher level of Interleukin 4 Induced 1 (IL4I1) compared to responders. IL4I1 is known to promote tumor cell proliferation and suppress immune responses, and it was observed to enhance immune suppression via the IDO1-AHR-Kyn metabolic pathway. In particular, the presence of IL4I1 inhibited the cytotoxicity of CAR-T cells in vitro, whereas the knockout of IL4I1 in M2 macrophages reversed their suppressive effect in vivo [113].

#### Monocytes in Mantle Cell Lymphoma

Monocytes play a significant role in the TME and clinical progression of MCL, and monocyte count may also hold prognostic value. In a retrospective study of 103 MCL cases, elevated AMC at diagnosis was associated with advanced-stage disease and poorer OS [114]. The monocyte-to-platelet ratio is another PB parameter that reflects the significance of monocytes in MCL. M-MDSC can promote circulating monocyte-platelet aggregates, reducing platelet counts. Alongside elevated LDH levels and BM involvement, a high monocyte-to-platelet ratio emerged as an independent predictor of poor outcome, highlighting its potential utility in future risk stratification models [115].

MCL cells actively recruit monocytes into the TME via tumor-derived soluble factors, with CCL3 as a key driver [116]. Recruitment is enhanced in aggressive or highly proliferative cases, reflected by increased CD14 and CD163 expression in lymph node and BM samples [117]. Within TME, MCL cells polarize monocyte-derived macrophages toward an M2-like phenotype that promotes cancer cell survival. In a xenograft model, co-injection of human MCL cells with CD14⁺ monocytes enhanced tumor growth, an effect reversed by macrophage depletion with liposomal clodronate [118].

## Role of Monocytes in Cytokine Release Syndrome and Neurotoxicity

Monocytes are recently recognized as key mediators of severe toxicities associated with cytotoxic T cell-based immunotherapies used in the treatment of hematologic malignancies. Cytokine release syndrome (CRS) is a severe, potentially life-threatening complication associated with immunotherapies involving cytotoxic T cells, particularly CAR-T cell therapy and, to a lesser extent, bispecific T-cell engagers (BiTEs). In addition to CRS, these immune therapies can also lead to immune effector cell-associated neurotoxicity syndrome (ICANS), a distinct but often overlapping complication characterized by neuroinflammation, which is similarly driven by cytokine-mediated disruption of the blood-brain barrier and activation of myeloid cells within the central nervous system. While T cells are the initiators of this immune activation, monocytes and monocyte-derived macrophages are now recognized as the primary effectors of CRS and ICANS pathophysiology.

Evidence from in vitro co-culture systems and mouse models demonstrates that monocytes and macrophages rather than CAR-T cells are the dominant source of IL-6, which is the key driver of CRS severity [119, 120]. The mechanism is believed to involve an initial wave of CAR-T cell-derived cytokines that activate monocytes. Following target tumor cell recognition, activated CAR-T cells release cytokines such as IFN-γ and GM-CSF, which in turn activate monocytes to secrete large quantities of IL-6, TNF-α, IL-1β, and CCL2. These cytokines mediate the systemic inflammation and associated symptoms of CRS, such as fever, hypotension, hypoxia, and organ dysfunction [101, 119].

The central role of IL-6 in driving CRS has led to the development of targeted strategies aimed at modulating its activity. IL-6 receptor blockade with tocilizumab remains the approved frontline treatment for CRS [121]. This therapy aims to reduce systemic toxicity without compromising the antitumor efficacy of CAR-T cells, highlighting the need to precisely manage monocyte responses to improve patient safety and treatment outcomes. While tocilizumab is not effective in mitigating CAR-mediated neurotoxicity, the prophylactic administration of anakinra—an interleukin-1 receptor antagonist—has shown promise in reducing the risk of ICANS without compromising CAR-T cell efficacy [122]. Another investigational strategy for CRS mitigation involves targeting monocytes directly. It was demonstrated that depletion of monocytes with clodronate liposomes significantly reduced CRS severity in leukemia-bearing humanized mice treated with CAR-T cells [119]. Additionally, CD44v6-directed CAR-T cells, which target both leukemia cells and monocytes, were shown to shorten the duration of CRS, although they did not fully prevent it [119]. While these findings underscore the central role of monocytes in CRS pathogenesis and suggest that monocyte depletion may be a viable therapeutic avenue, further studies are required to validate the safety, feasibility, and efficacy of this approach.

## Conclusions

In summary, monocytes and their progeny are dynamic, key components of the TME in B-cell malignancies. High-dimensional phenotyping and transcriptomics revealed their remarkable heterogeneity and plasticity, which renders their identification and characterization particularly challenging, especially in the context of cancer. Increased monocyte counts—particularly of non-classical subsets in peripheral blood and lymphoid tissues—have been observed across B-ALL, CLL, and B-NHL. Far from passive bystanders, monocytes actively shape tumor progression, immune evasion, and therapeutic responses, primarily through differentiation into immunosuppressive macrophages and dendritic cells. Their prognostic relevance is reflected in peripheral blood indices, functional states, and transcriptional signatures, with elevated levels predicting poorer outcomes and reduced responses to therapies, including small-molecule inhibitors and CAR-T cell therapy. Over the past decade, studies have further clarified the mechanisms of monocyte trafficking to the TME and their immunosuppressive activities, while also uncovering a “dark side” of monocytes as central mediators of severe immunotherapy-related toxicities, including CRS and ICANS. These insights suggest that therapeutic strategies targeting monocytes—through inhibition of recruitment, depletion or reprogramming of suppressive subsets—may improve both efficacy and safety. However, these approaches are still in the early stages of investigation and further research is needed to optimize and develop more effective and feasible myeloid-directed therapies. Integrating such strategies into current and emerging treatment regimens thus holds promise for improving outcomes in B-cell malignancies.

## Key References

Of outstanding importance.

- Contreras Yametti GP, Evensen NA, Schloss JM, Aldebert C, Duan E, Zhang Y, et al. Flow cytometric assessment of leukemia-associated monocytes in childhood B-cell acute lymphoblastic leukemia outcome. Blood Adv 2023;7:3928–31.10.1182/bloodadvances.2023010044.
  - This study demonstrates that monocyte populations are altered in pediatric B-ALL and that their levels are associated with treatment outcomes.
- Guo Y, Pei H, Lu B, Zhang D, Zhao Y, Wu F, et al. Aberrantly expressed Wnt5a in nurse-like cells drives resistance to Venetoclax in chronic lymphocytic leukemia. Cell Death Discov 2022;8:82. 10.1038/s41420-022-00884-y.
  - This study shows that monocyte-derived cells contribute to therapy resistance in CLL.
- Mozas P, Ould Ammar R, Chartier L, Nastoupil L, Bachy E, Bezsera SM, et al. A low lymphocyte-to-monocyte ratio is independently associated with early relapse (POD24) in high tumour burden follicular lymphoma: A RELEVANCE subanalysis. Br J Haematol 2025;206:1380–9. 10.1111/bjh.20038.
  - This study shows that low lymphocyte-to-monocyte ratio is associated with survival and progression risk in FL.
- Carniti C, Caldarelli NM, Agnelli L, Torelli T, Ljevar S, Jonnalagadda S, et al. Monocytes in leukapheresis products affect the outcome of CD19-targeted CAR T-cell therapy in patients with lymphoma. Blood Adv 2024;8:1968–80.10.1182/bloodadvances.2024012563.
  - This study highlights that monocytes negatively impact CAR T-cell therapy in relapsed/refractory large B-cell lymphoma, with high counts predicting poor response and survival.
- Guan X, Wang Y, Fang T, Wang J, Li R, Hao M, et al. Lymphoma cell-driven IL-16 is expressed in activated B-cell-like diffuse large B-cell lymphomas and regulates the pro-tumor microenvironment. Haematologica 2025;110:425–38. 10.3324/haematol.2024.285304.
  - This study shows that tumor cells recruit monocytes to the DLBCL tumor microenvironment, where they differentiate into macrophages and promote tumor growth.
- Le K, Sun J, Ghaemmaghami J, Smith MR, Ip WKE, Phillips T, et al. Blockade of CCR1 induces a phenotypic shift in macrophages and triggers a favorable antilymphoma activity. Blood Adv 2023;7:3952–67.10.1182/bloodadvances.2022008722.
  - This study shows that monocyte recruitment and macrophage polarization in MCL promote tumor growth, and that reprogramming macrophages to an immunogenic phenotype can suppress lymphoma progression.
- Park JH, Nath K, Devlin SM, Sauter CS, Palomba ML, Shah G, et al. CD19 CAR T-cell therapy and prophylactic anakinra in relapsed or refractory lymphoma: phase 2 trial interim results. Nat Med 2023;29:1710–7. 10.1038/s41591-023-02404-6.
  - This study highlights the role of monocyte-derived factors in CAR T-cell–related neurotoxicity in B-cell lymphomas and suggests therapeutic strategies, such as anakinra, to prevent it.

Of importance

- Liu L, Yu X, Li Z, He X, Zha J, Lin Z, et al. Revealing the evolution of the tumor immune microenvironment in follicular lymphoma patients progressing within 24 months using single-cell imaging mass cytometry. J Hematol OncolJ Hematol Oncol 2022;15:115.https://doi.org/10.1186/s13045-022-01326-z.
  - This study highlights the role of macrophages in reshaping the FL tumor microenvironment, aiding malignant cell immune evasion and disease progression.
- Zöphel S, Küchler N, Jansky J, Hoxha C, Schäfer G, Weise JJ, et al. CD16+ as predictive marker for early relapse in aggressive B-NHL/DLBCL patients. Mol Cancer 2024;23:210.https://doi.org/10.1186/s12943-024-02123-7.
  - This study shows that altered monocyte populations are associated with higher relapse risk in aggressive B-cell lymphoma, highlighting their prognostic significance.
- Cui N, Leary P, Ivanova V-S, Stirm K, Kirsche L, Aceto N, et al. PDL1-expressing macrophages infiltrate diffuse large B-cell lymphoma and promote lymphoma growth in a MYC-driven experimental model. Blood Cancer J 2025;15:66. https://doi.org/10.1038/s41408-025-01281-1.
  - This study shows that macrophages dominate the DLBCL tumor microenvironment, suppress immune control, and represent a key target for immunotherapy.
- Zhang R, Zhang Y, Xiao H, Liu Q, Zhao M. Knockout IL4I1 affects macrophages to improve poor efficacy of CD19 CAR-T combined with PD-1 inhibitor in relapsed/refractory diffuse large B-cell lymphoma. J Transl Med 2025;23:105. https://doi.org/10.1186/s12967-024-06028-3.
  - This study shows that macrophages limit CAR-T cell efficacy in DLBCL, highlighting these cells as therapeutic targets.

## Data Availability

No datasets were generated or analysed during the current study.
